# Case report: A case of brucellosis misdiagnosed as coronavirus disease 2019/influenza in China

**DOI:** 10.3389/fpubh.2023.1186800

**Published:** 2023-09-01

**Authors:** Shuai Qin, Dongyue Lv, Ran Duan, Xiaojin Zheng, Asaiti Bukai, Xinmin Lu, Qun Duan, Mingrun Yu, Huaiqi Jing, Xin Wang

**Affiliations:** ^1^State Key Laboratory of Infectious Disease Prevention and Control, National Institute for Communicable Disease Control and Prevention, Chinese Center for Disease Control and Prevention, Beijing, China; ^2^Akesai Kazak Autonomous County Center for Disease Control and Prevention, Jiuquan, China; ^3^Taizhou Center for Disease Control and Prevention, Taizhou, China

**Keywords:** brucellosis, coronavirus disease 2019, misdiagnosis, erythrocyte sedimentation rate, febrile diseases

## Abstract

Brucellosis is an important zoonosis and a multisystem disease. The signs and symptoms of brucellosis are not specific. In the clinical, brucellosis is often ignored and misdiagnosed. We report a case of brucellosis who was misdiagnosed as coronavirus disease 2019 (COVID-19)/influenza and received delayed treatment during strict COVID-19 control. The neglect of other diseases due to COVID-19 and empirical diagnosis and treatment by medical staff are part of the reasons for misdiagnosis. Otherwise, the normal erythrocyte sedimentation rate (ESR), increased white blood cell count (WBC), and increased neutrophil count (NEUT) of this patient was also a cause of misdiagnosis, which is an important reminder for diagnosis. For patients with the unknown origin of fever and other symptoms related to brucellosis, especially those from endemic areas of brucellosis, brucellosis screening is a priority item, and grassroots doctors should be vigilant and standardize the diagnosis and treatment based on epidemiology history, clinical manifestation, and laboratory tests according to the diagnostic criteria of brucellosis.

## Introduction

Brucellosis is an important worldwide zoonosis. Human infections are primarily acquired through contact with infected animals and their secretions (1, 2). Human-to-human transmission takes place through blood transfusion, bone marrow transplantation, and mother-to-fetus transmission (3). The signs and symptoms of brucellosis are not specific, fever, sweat, fatigue, and joint ache are the most common manifestation of human brucellosis, these are similar to influenza, severe colds, coronavirus disease 2019 (COVID-19), malaria, and other infectious diseases, so clinical diagnosis is difficult in place of lack of health facility and specific and rapid diagnostic methods (4, 5). An epidemiological survey based on 2060 cases collected from brucellosis clinics in China showed that 57.62% of patients were misdiagnosed or suspected of having other diseases with similar clinical symptoms (6). Other report indicate that brucellosis is easily misdiagnosed as a variety of other infections and noninfectious diseases (7). In clinical set up, diagnosis of brucellosis is made based on history, clinical manifestation, and laboratory tests including culture, serological tests, and nucleic acid amplification assays. Besides, the hematological parameters including biochemical examination and blood routine examination are commonly observed in the diagnosis of brucellosis (8–11).

Because of the COVID-19 pandemic, China has taken strict measures to control patients with fever. It is crucial for the prognosis to identify non-COVID-19 infections in patients with fever as early as possible. If patients with brucellosis fail to receive timely and standardized treatment, the probability of cure will be greatly reduced (12). The disease is more likely to progress to a chronically incapacitating disease with severe complications, which affect patients’ working ability and life quality (13). Here, we report a *Brucella* case misdiagnosed as COVID-19/influenza. Overly strict management of COVID-19, neglect of normal erythrocyte sedimentation rate (ESR), and increased white blood cell count (WBC) and neutrophil count (NEUT) of the patient caused the misdiagnosis of brucellosis, which is a warning for the diagnosis and treatment of brucellosis amidst the pandemic.

## Case presentation

On October 5, 2022, a 61 years-old male patient was presented to the local hospital following a one-week history of fever [38.5°C(101.3°F)], systemic muscle and joint pain, and burning sensation in the skin. The patient came from a remote county in the Altun Mountain region of Gansu province in western China, which is a brucellosis endemic area. A survey on the epidemic of brucellosis in this area showed that the brucella seroprevalence in livestock was 4.2%, and that of human population was 1.2% (14). The hospital treated him as a suspected case of COVID-19. Between October 8 and October 11, he tested negative for COVID-19 on 4 consecutive days, based on the quantitative polymerase-chain-reaction (qPCR) test at the local center for disease control and prevention. Subsequently, he self-administered a herbal medicine Ganmaoling granule which consists of eight main ingredients: Ye Ju Hua (Flos *Chrysanthemi indici*), Jin Zhan Yin Pan (*Bidens biternata* Merr. et Sherff), Gang Mei (Radix *Ilex asprella*), San Cha Ku (Radix *Evodia lepta*), caffeine, acetaminophen, chlorpheniramine maleate, and menthol oil. Although the fever was temporarily alleviated, other symptoms worsened. On October 18, the patient went to the local hospital for treatment, and the COVID qPCR was again negative, but his white blood cell count and other blood indicators increased, indicating serious infection (Table 1). The patient was diagnosed with influenza with bacterial infection without any pathogenic or serological examination, and was clinically treated with metamizole sodium, intramuscular injection of penicillin, and oral sulfanilamide. Thereafter, the symptoms were slightly relieved. However, for nearly a month from October 25 onwards, he started experiencing sleep hyperhidrosis and had a fever (98.6–100.4°F) from 4 to 5 AM every day. After oral metamizole sodium, the temperature returned to normal, but the patient continued to feel ill. After further inquiry, it was understood that the patient raised cattle and sheep infected with *Brucella melitensis.* In addition, a sheep had a miscarriage, which was confirmed to be infected with *B. melitensis*, and the patient handled the aborted animal without personal protection (15, 16). No one else contact with the infected animals and aborted foetus, there was no confirmed case of brucellosis in his family members and neighbors.

**Table 1:** Important indicators of the patient’s clinical examination.

Indicator	2022.10.18	2022.11.25	2022.11.26	Reference range	Unit
WBC	18.03^*^	8.66	—	4–10	×10^9^/L
NEUT#	13.17^*^	5.11	—	2–7	×10^9^/L
LYMPH#	3.46	3.16	—	0.8–4	×10^9^/L
MONO#	1.39^*^	0.30	—	0.12–1.2	×10^9^/L
EO#	0.00^*^	0.08	—	0.02–0.5	×10^9^/L
BASO#	0.01	0.01	—	0–0.1	×10^9^/L
IG#	0.21^*^	0.05^*^	—	0	×10^9^/L
NEUT%	73^*^	59	—	50–70	%
LYMPH%	19.2^*^	36.5	—	20–40	%
MONO%	7.7	3.5	—	3–10	%
EO%	0.0^*^	0.9	—	0.5–5	%
BASO%	0.1	0.1	—	0–1	%
IG%	1.2^*^	0.5^*^	—	0	%
RBC	4.57	5.42	—	3.5–5.5	×10^12^/L
HGB	158	175^*^	—	120–170	g/L
RDW-CV	12.4	13.0	—	11–16	%
RDW-SD	42.8	43.7	—	35–56	fL
PLT	202	131	—	100–300	×10^9^/L
MPV	11.9	11.3	—	6.5–12	fL
PDW	16	16.7	—	15–17	
CRP	57.8^*^	37.5^*^	—	0–10	mg/L
ESR	6	—	4	0–15	mm/h
hsCRP	>5^*^	—	—	0–3	mg/L
Anti-CCP	48.3^*^	—	—	0–45	U/mL

^*^Abnormal indicators. —Indicates that testing was not done. Indicator interpretation: WBC, white blood cell count; NEUT#, neutrophil count; LYMPH#, lymphocyte count; MONO#, monocyte count; EO#, eosinophil count; BASO#, basophil count; IG#, immature granulocyte count; NEUT%, neutrophil percentage; LYMPH%, lymphocyte percentage; MONO%, monocyte percentage; EO%, eosinophil percentage; BASO%, basophil percentage; IG%, immature granulocyte percentage; RBC, red blood cell count; HGB, haemoglobin; RDW-CV, coefficient variation of red cell distribution width, RDW-SD, standard deviation in red cell distribution width; PLT, platelet count; MPV, mean platelet volume; PDW, platelet distribution width; CRP, c-reactive protein; ESR, erythrocyte sedimentation rate; hsCRP, hypersensitive c-reactive protein; anti-CCP, anti-cyclic peptide containing citrulline.

**Figure 1:**
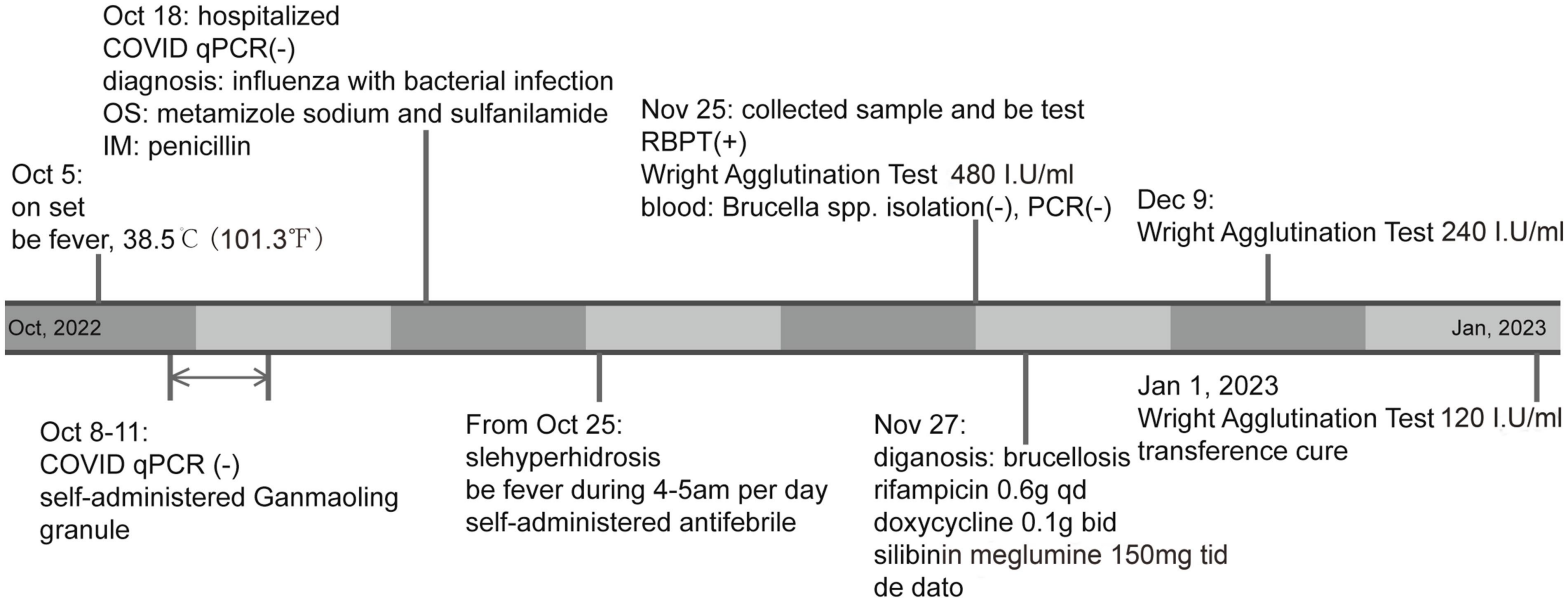
Patient symptoms, examinations, and treatment process.

On November 25 the Rose-Bengal plate test (RBPT) of the patient’s serum was positive. The *Brucella* serum antibodies titer was tested by Wright agglutination test, and the result was 480 I.U/mL (17). Data from routine surveillance for brucellosis showed that the patient’s serum antibody test for brucellosis was negative on 28 July. But the blood culture and PCR tests for *Brucella* were negative on 25 November. As local patients are generally unwilling to undergo bone marrow puncture, and the hospital is located in a remote area of China with limited medical level, doctors lack experience in bone marrow puncture, so bone marrow culture is not performed. To avoid the *Yersinia enterocolitica* serotype O:9 which may lead to cross-reactivity in serology, the slide agglutination tests were performed. The results of sera collected from patients at different times were all negative, which ruled out the possibility of *Y. enterocolitica* O:9 infection (18). Although the blood indicators at this time tended to be normal compared with those obtained a month before (Table 1), the patient still experienced muscle and joint pain, and was finally diagnosed with brucellosis infection case which defined as a patient with a history of epidemiologic exposure and associated clinical manifestations of brucellosis, and the test result of Wright agglutination test ≥60 I.U/mL. On November 27, the patient received specific treatment for brucellosis: rifampicin 0.6 g (qd), doxycycline 0.1 g (bid), and silibinin meglumine 150 mg (tid). Rifampicin and doxycycline are both hepatotoxic, while silibinin meglumine has the effect of protecting the liver, so the above three drugs are used simultaneously for treatment. After 1 week of treatment, his condition improved significantly. After 2 weeks of treatment, the Wright agglutination test result had reduced to 240 I.U/mL. After 35 days of treatment, that result was 120 I.U/mL, the symptoms completely disappeared, and the treatment was stopped (Figure 1).

## Discussion

To control the spread of the pandemic, COVID-19 has been tightly managed in China until December 2022. In clinical practice, patients with fever were treated as suspected cases of COVID-19. After excluding COVID-19, they were admitted to hospitals for routine treatment, leading to misdiagnosis of some diseases or delayed treatment, such as the pneumonic plague case in Tibet, China, in September 2022 (19). Plague infection wasn’t considered in this case, as there was no animal surveillance of plague and no human cases historically in the area. Further, COVID-19 was prevalent in Xigaze, and the hospital only conducted multiple COVID-19 examinations, resulting in delayed treatment and eventual death.

The delayed treatment of the brucellosis case we reported was also caused by misdiagnosis. COVID-19 infection case was defined as a person who meets the clinical manifestations of COVID-19 and the epidemiological history of COVID-19, and tests positive for COVID-19 nucleic acid or antibodies. However, doctors are worried about the spread of the epidemic due to missed diagnosis of COVID-19, so they give priority to treating the patients with fever as COVID-19 infection case and perform multiple tests on the patient without considering the screening of other diseases. Because of the misdiagnosis as COVID-19 and influenza with bacterial infection, the treatment was delayed by nearly 1 month. The diagnosis of influenza with bacterial infection was made empirically. The patient was a herdsman with an obvious epidemiological history. However, because of the lack of basic training in the diagnosis and treatment of infectious diseases, the hospital did not consider infectious diseases and did not query the epidemiological history, leading to misdiagnosis and delayed treatment. The misdiagnosed case of brucellosis is the only case we have collected so far. We found many brucellosis patients in the area, but other patients will voluntarily state the history of brucellosis exposure during the visit, and the doctor will make a differential diagnosis of brucellosis. The patient lives in a region where animal brucellosis is relatively severe. Clinicians should consider brucellosis in patients with unexplained fever, based on their epidemiological history. Empirical treatment with penicillin and sulfanilamide failed to achieve optimal results. Penicillin is ineffective against *Brucella* (20). Sulfanilamide, as a chemical drug, has a certain antibacterial effect on *Brucella*, but it cannot be effectively treated, leading to continued disease development (21).

The hematological parameters including biochemical examination and blood routine examination are commonly observed in the diagnosis of brucellosis. Our patient’s ESR was normal, and the patient has increased WBC and NEUT (Table 1), which could also be a reason for misdiagnosis and an important point to remember for future clinical diagnosis of brucellosis. As the highest titer of IgG antibody is produced at around 20 days, it can be seen from the infection process of this patient that his highest Wright agglutination test result was at least 960 I.U/mL. The failure to isolate the pathogen and the negative PCR test in this patient were closely related to the misdiagnosis, and the opportunity for pathogenic diagnosis was lost because of misdiagnosis. Therefore, after excluding other microbial infections, clinicians should make a comprehensive judgment based on epidemiological history, clinical symptoms, and laboratory examinations; carry out immunological examination; and confirm the diagnosis and start treatment for suspected brucellosis cases as soon as possible to improve the treatment effect and avoid acute brucellosis turning into chronic brucellosis.

Currently, COVID-19 and influenza are still prevalent. COVID-19, influenza, and brucellosis have similar clinical symptoms. These three diseases are easy to be misdiagnosed. Other countries have also reported cases of brucellosis misdiagnosed as COVID-19 (22, 23). Those cases also presented symptoms of fever, fatigue and arthralgia and the case 2 reported by Salman et al. was also with a normal ESR (Table 2). Similar to our case, the misdiagnosis or delayed treatment of these two cases were related to the failure to inquire about the epidemic contact history in time. Therefore, in brucellosis endemic areas, the contact history of brucellosis should be confirmed as soon as possible for patients with fever. The possibility of brucellosis should be considered for patients with epidemiology history of contact with infected animals or ingestion of infected meat or unpasteurized dairy products. In addition, the spleen examination of these two cases was abnormal, which also suggested that liver and spleen pathological examination was necessary for the patients with fever to assist in differential diagnosis, and equally important, blood culture should be carried out in time before drug intervention. During the epidemic period of COVID-19 and influenza, patients with fever take antibiotics empirically, which affects the isolation of pathogenic bacteria. The ESR results of the twice tests in this case were normal. Other studies have also reported that patients with brucellosis have normal ESR. Whether this is related to the medication taken by the patients needs further study. This phenomenon suggests that we should pay attention to the influence of drugs on hematological parameters in clinical diagnosis to prevent misdiagnosis due to empirical diagnosis. For patients with the unknown origin of fever and other symptoms related to brucellosis, especially those from endemic areas of brucellosis, brucellosis screening is a priority item, and grassroots doctors should be vigilant and standardize the diagnosis and treatment based on epidemiology history, clinical manifestation, and laboratory tests according to the diagnostic criteria of brucellosis. In addition, complete serological monitoring was performed from the acute phase to recovery for our case. Changes in serologic titers in this patient demonstrate the importance of timely and specific treatment of brucellosis. It was also convenient to grasp the condition for timely adjustment of treatment. This suggests that patients with brucellosis should continue to follow up with the same physician to prevent delay in diagnosis and treatment.

**Table 2:** Symptoms and laboratory examination results of case of brucellosis misdiagnosed as coronavirus disease 2019.

	Our case	Case 1	Case 2	Reference range	Unit
*Symptoms*					
Fever	Yes	Yes	Yes	—	—
Fatigue	Yes	Yes	Yes	—	—
Arthralgia	Yes	Yes	Yes	—	—
Sweat	Yes	No	No	—	—
*Laboratory examination*					
WBC	18.03^*^/8.66	4.5	4.8	4–10	×10^9^/L
HGB	158/175^*^	124	142	120–170	g/L
NEUT#	13.17^*^/5.11	—	1.6^*^	2–7	×10^9^/L
LYMPH#	3.46/3.16	—	2.9	0.8–4	×10^9^/L
PLT	202/131	89^*^	263	100–300	×10^9^/L
CRP	57.8^*^/37.5^*^	66.54^*^	63.95^*^	0–10	mg/L
ESR	6/4	—	10	0–15	mm/h

Case number: Case 1: reported by Kucuk and Gorgun. Case 2: reported by Salman et al. ^*^Abnormal indicators. —Indicates that testing was not done. Indicator interpretation: WBC, white blood cell count; HGB, haemoglobin; NEUT#, neutrophil count; LYMPH#, lymphocyte count; PLT, platelet count; CRP, c-reactive protein; ESR, erythrocyte sedimentation rate.

## Conclusion

We report a misdiagnosed case of brucellosis. During strict COVID-19 control, some diseases were misdiagnosed or received delayed treatment. The normal ESR, increased WBC, and increased NEUT was also a cause of misdiagnosis in this case, which is an important reminder for diagnosis. For patients with the unknown origin of fever and other symptoms related to brucellosis, especially those from endemic areas of brucellosis, brucellosis screening is a priority item, and grassroots doctors should be vigilant and standardize the diagnosis and treatment based on epidemiology history, clinical manifestation, and laboratory tests according to the diagnostic criteria of brucellosis. A better understanding of the clinical significance of hematological parameters and timely improvement of the level of pathogen detection can facilitate early diagnosis and prevent misdiagnosis of brucellosis. Brucellosis patients should be educated to visit medical specialists rather than paramedics and continue follow-up with the same physician to prevent delay in diagnosis, or unnecessary or wrong treatment, with complication of the unmanaged disease.

## Data availability statement

The original contributions presented in the study are included in the article/supplementary material, further inquiries can be directed to the corresponding author.

## Ethics statement

The studies involving human participants were reviewed and approved by the ethics committee of the National Institute for Communicable Disease Control and Prevention of the Chinese Center for Disease Control and Prevention. The patients/participants provided their written informed consent to participate in this study. Written informed consent was obtained from the individual(s) for the publication of any potentially identifiable images or data included in this article.

## Author contributions

XW contributed to the conception, design of the work, and supervised the work. SQ, RD, and QD performed the experiments. XZ, AB, and XL provided data of patient. SQ, DL, HJ, and MY performed the analysis and interpretation of the data. SQ, DL, and RD drafted the manuscript. XW and XZ reviewed and critically revised the manuscript. All authors contributed to the article and approved the submitted version.

## Funding

This work was supported by the National Key Research and Development Program of China (2022YFC2602203).

## Conflict of interest

The authors declare that the research was conducted in the absence of any commercial or financial relationships that could be construed as a potential conflict of interest.

The reviewer ZL declared a shared affiliation with the authors SQ, DL, RD, QD, HJ, and XW to the handling editor at the time of review.

## Publisher’s note

All claims expressed in this article are solely those of the authors and do not necessarily represent those of their affiliated organizations, or those of the publisher, the editors and the reviewers. Any product that may be evaluated in this article, or claim that may be made by its manufacturer, is not guaranteed or endorsed by the publisher.
